# Effect of foreknowledge on neural activity of primary “go” responses relates to response stopping and switching

**DOI:** 10.3389/fnhum.2015.00034

**Published:** 2015-02-04

**Authors:** Benjamin Xu, Sarah Levy, John Butman, Dzung Pham, Leonardo G. Cohen, Marco Sandrini

**Affiliations:** ^1^Human Cortical Physiology and Neurorehabilitation Section, The National Institute of Neurological Disorders and Stroke, The National Institutes of HealthBethesda, MD, USA; ^2^Center for Neuroscience and Regenerative MedicineBethesda, MD, USA; ^3^Clinical Center, Department of Radiology, National Institutes of HealthBethesda, MD, USA

**Keywords:** response inhibition, foreknowledge, fMRI, stop-signal, impulse

## Abstract

Being able to stop (or inhibit) an action rapidly as in a stop-signal task (SST) is an essential human ability. Previous studies showed that when a pre-stimulus cue warned of the possible need to stop a response in an upcoming trial, participants’ response time (RT) increased if the subsequent trial required a “go” response (i.e., “go” RT cost) relative to a trial where this uncertainty was not present. This increase of the “go” RT correlated with more efficient response stopping. However, it remains a question whether foreknowledge of upcoming inhibition trials given prior to the task is sufficient to modulate neural activity associated with the primary “go” responses irrespective of whether stopping an overt response is required. We presented three task conditions with identical primary (i.e., “go”) response trials but without pre-stimulus cues. Participants were informed that Condition 1 had only “go” trials (All-go condition), Condition 2 required a “stop” response for some trials (Stop condition), and Condition 3 required a response incongruent with the primary response (i.e., Switch response) for some trials (Switch condition). Participants performed the tasks during functional magnetic resonance imaging (fMRI) scans. Results showed a significant increase in the “go” RT (cost) in the Stop and Switch conditions relative to the All-go condition. The “go” RT cost was correlated with decreased inhibition time. fMRI activation in the frontal-basal-ganglia regions during the “go” responses in the Stop and Switch conditions was also correlated with the efficiency of Stop and Switch responses. These results suggest that foreknowledge prior to the task is sufficient to influence neural activity associated with the primary response and modulate inhibition efficiency, irrespective of whether stopping an overt response is required.

## Introduction

The ability to voluntarily stop or change an on-going action rapidly is an important part of human cognitive capacity (Logan et al., 1984). It is the ability of our system to make an instantaneous/reactive response (within a fraction of a second) to terminate (inhibit) a planned or an on-going action when an undesirable change in the environment suddenly occurs. Converging evidence from behavioral, psychophysiological, electrophysiological, clinical neuroscience, brain imaging, and noninvasive brain stimulation studies has outlined specific brain regions in the right inferior frontal cortex (rIFC), dorsal medial frontal areas, and the basal ganglia (i.e., the frontal-basal-ganglia (FBG) network) that are critical for rapid response inhibition (Band and van Boxtel, 1999; Miller and Cohen, 2001; Sumner et al., 2007; Chambers et al., 2009; Aron, 2011; Juan and Muggleton, 2012). However, how such rapid response inhibition is achieved and the neural mechanism(s) underlying it remain important research questions.

A common method for understanding the mechanism(s) underlying rapid response inhibition is to apply the stop-signal task (SST; Logan and Cowan, 1984). In a typical SST, the majority of the trials require a rapid response (i.e., the primary “go” response). Only for a small number of trials, subjects are signaled to withhold/terminate their response (i.e., the Stop response) when a stop-signal appears following a variable delay time after the onset of a “go” stimulus (i.e., stop-signal delay or SSD). The SSD, the distribution of the “go” response time (RT), and the probability of making a stop response are used to estimate the stop-signal response time (SSRT), an index of the time needed to stop a response (Logan et al., 1984; Verbruggen and Logan, 2008). Recent studies showed if participants were given foreknowledge such as a pre-stimulus or a within-stimulus cue on a trial-by-trial basis indicating that a Stop response may be required in the upcoming trial, their RT increased if the subsequent trial required a “go” response comparing to when a pre-stimulus cue indicating a definitive “go” response (Chikazoe et al., 2009; Claffey et al., 2010; Jahfari et al., 2010; Cai et al., 2011). Chikazoe et al. (2009) also reported that the increase in the RT of the “go” response immediately preceding a successfully stopped response (or a stop-inhibit response) was associated with a reduction in the SSRT, indicating more efficient response inhibition (also see Jahfari et al., 2010). In addition, these studies showed that functional magnetic resonance imaging (fMRI) activation in the rIFC, pre-supplementary motor area (pre-SMA), and subcortical regions was greater for the “go” responses in conditions where stopping trials were present. Findings from these studies suggest that the response-specific cue (or foreknowledge) induces a “go” response cost that benefits rapid stopping in a SST.

Studies using electrophysiological recording in the basal ganglia in non-human primates also indicated that the mere expectation of an upcoming event or a stopping response influenced neural activity associated with the expected response (Apicella et al., 1992; Schultz et al., 1992). Other studies using transcranial magnetic stimulation showed that when participants were instructed that an upcoming trial may require a Stop response, neural excitability in the primary motor cortex (M1) corresponding to the responding hand was significantly reduced prior to the onset of the stimulus (Claffey et al., 2010; Jahfari et al., 2010; Cai et al., 2011). Greenhouse et al. (2012) reported that selective suppression of the excitability in the motor cortex was observed only when the subjects showed significant slowing of the primary responses (i.e., a large “go” response cost). It is also well known that preparing or anticipating to make a response to a target changes neural activity (e.g., stimulus preceding negativity and rapid neuronal phase synchronization) associated with critical components of the action process such as attention, action plan, and execution (Brunia, 1993; Brunia and van Boxtel, 2001; Gross et al., 2006; Zandbelt and Vink, 2010; Swann et al., 2013). Findings from these studies are evidence that goal-directed top-down signals modulate task/target relevant neural responses (Kanwisher and Wojciulik, 2000; Kastner and Ungerleider, 2000; Corbetta and Shulman, 2002).

However, studies on the effect of foreknowledge on neural activity in rapid response inhibition in humans are limited. The few reported studies focused primarily on stopping/termination of an overt motor response and mostly with short blocks of trials or with trial-by-trial cues that could have encouraged the readiness to stop an upcoming response. More evidence is needed to determine whether foreknowledge influences the response inhibition process directly (Verbruggen and Logan, 2009; Greenhouse and Wessel, 2013). The current study addressed two questions: (1) whether foreknowledge of the presence of inhibition trials^1^ given before performing the task would be sufficient to modify neural activity associated with the primary response even when no pre-stimulus cues are provided during the task and; (2) whether neural activity within the FBG network during the “go” response would be predictive of the efficiency of stopping an on-going motor response or stopping an impulse/tendency for the primary response without terminating a motor response altogether.

## Material and methods

### Participants

Eighteen healthy volunteers (9 males and 9 females, mean age = 26.4 ± 4.5) participated in the study. All participants had a normal structural MRI, neurological examination, with normal or corrected vision, and were right-handed based on the evaluation with the Edinburgh Handedness Inventory (Oldfield, 1971). They all gave a signed written consent approved by the Combined Neuroscience Institutional Review Board at the National Institutes of Health for participating in the study and in accordance with the Declaration of Helsinki. Participants received monetary compensation for their time in the study.

### Task materials and procedure

**Figure 1:**
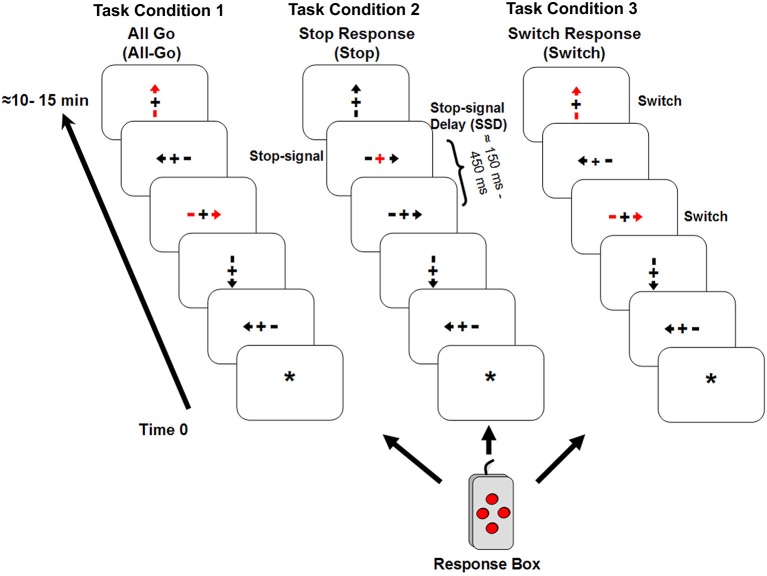
**It shows the three experimental task conditions**. Condition 1 (All-Go) had only “go” trials; Condition 2 (Stop) had 25% stop-signal trials in addition to the “go” trials; and Condition 3 (Switch) had 25% of Switch trials that required subjects to press the opposite button when a red arrow appeared. Similar to Condition 2, the remaining trials in Condition 3 were the “go” trials.

Participants performed the tasks (described below) during an event-related fMRI (efMRI) session. The stimuli consisted of four orientations (up, down, left, and right) of an arrow with a fixation point “+” in the middle (see Figure 1). All four orientations had equal probability of occurrence in all task conditions (see below). The arrow stimuli were presented one at a time using the E-Prime software (by Psychological Software Tools, Inc) for a duration of 1500 ms or until a response was made. The data collection period was 2000 ms for each trial. The stimulus dimension was maintained at less than 2 degrees of visual angle relative to the subject’s viewing position inside the MR scanner. The inter-stimulus-interval (ISI) was jittered for the efMRI design with an average ISI of about 4 s (range 2–6 s) yielding a stimulus-onset-asynchrony (SOA) between 3.5 and 7.5 s. There were also six 10 s ISIs interspersed within each scan run. Prior to the first stimulus onset, there was a 10 s resting period with a yellow star “*” as the fixation point in the center of the display. At the offset of each stimulus, the fixation point appeared and stayed on until the onset of the next trial (the 10 s interval was indicated with an uppercase letter “R”). A four-button response box (see Figure 1) was configured such that the top, bottom, left, and right buttons corresponded to the four stimulus orientations. The response buttons were situated in equal distance around a center space (about 1 cm^2^). All responses were made with the right index finger. The primary response (i.e., the “go” response) required pressing the button consistent with the arrow orientation. Participants were instructed to always place their right index finger in the center space on the box between responses. They performed the task in three experimental conditions with identical “go” responses.

Task Condition 1 (All-Go condition): This condition included only the “go” responses (i.e., All-Go) with a total of 80 trials. All but 20 trials (25%) appeared in red color and the rest in gray. Participants were told explicitly that color was irrelevant for this task condition and that they should press the corresponding button as quickly as possible without sacrificing response speed for accuracy. The All-Go condition served three purposes: (1) to provide a baseline measure of the primary “go” response performance (i.e., RT and accuracy) when only “go” responses were required; (2) to establish a consistent stimulus-response association for the context of the response Switch condition (described below); and (3) to serve as a baseline for estimating the “go” RT cost (i.e., percent change or increase in RT at the presence of inhibition trials) in the Stop and Switch conditions and for adjusting the estimate of SSRT in the Stop Condition (see explanation below). All things being equal, the “go” RT cost reflects “proactive” slowing of the “go” responses in anticipation for a Stop or Switch response (Chikazoe et al., 2009; Zandbelt et al., 2013). However, if the “go” RT cost simply reflects an additional processing demand (i.e., Stop or Switch on some trials) but unrelated to the inhibition time, it should remain uncorrelated with the SSRT as it has typically been observed with the “go” RT (Logan et al., 1984). Similarly, its relationship (i.e., correlation coefficient) with the Switch cost (see explanation below) should also remain relatively unchanged comparing to its correlation with the “go” RT in the Switch condition.

Task Condition 2 (Stop condition): The Stop condition was a variant of the SST. It included a delayed visual-cue, i.e., the stop-signal, to indicate a Stop response. The primary “go” trials were identical to Condition 1 (All-Go) except that no red arrows were presented and for 40 (25%) out of the 160 trials, the “+” sign in the middle of the stimulus would turn red (the stop-signal) with a variable stop-signal delay (SSD) after the onset of the stimulus. Participants were instructed to withhold/stop their response as soon as the stop-signal appeared. The SSD was set at 150 ms for the first Stop trial and, then a staircase tracking method (Verbruggen and Logan, 2008) was implemented such that for every successfully-stopped (i.e., Stop-inhibit) response, the SSD was increased by 50 ms to make it harder to stop on the next trial, and for each fail-to-stop (i.e., Stop-respond) trial, the SSD decreased by 50 ms. The longest possible SSD was 450 ms. Participants were told explicitly at the beginning of the task condition that only a few trials would have the stop-signal and that they would not be able to withhold the response for many of those trials and that was expected. As it was for the All-Go condition, participants were instructed repeatedly that they should respond as quickly as possible for all trials and should not sacrifice response speed for accuracy. The dynamic SSD control method resulted in an overall Stop-inhibit rate of 57% (SD = 13), which was close to the ideal rate of 50% (Verbruggen and Logan, 2008). In addition, a subset of nine participants who had an average Stop-inhibit rate of 49.4% (SD = 5.6) was also analyzed separately.

Task Condition 3 (Switch condition): This condition had identical stimuli as those in Condition 1 (All-Go). The only difference was that participants were told to press the opposite response button (i.e., a Switch response) relative to the stimulus orientation when they saw a red arrow (40 out of 160 trials). For example, participants were instructed to press the left button when a right-pointing red arrow appeared. This manipulation placed the burden on stopping an impulse/tendency for the primary “go” response in order to initiate a Switch response. It is important to emphasize that as it was designed, the response component of the Switch trials was identical to that of the primary “go” responses and with the same extent of practice (or over-learning) in pressing the four response buttons. What differed was the need to inhibit/stop the primary but undesirable “go” process in the Switch condition (SwGo) and generate a Switch response.

The current design used identical button-press responses between task conditions. The key difference between the Stop and the Switch condition was that the Stop condition required the termination of an overt on-going motor response, while the Switch condition required the termination of an impulse/tendency for the primary “go” response without terminating a motor response altogether. The Switch condition in this study differed from a previous study that included a delayed cue and a different response key for the switch responses (Kenner et al., 2010). Kenner et al. (2010) reported that response switching engaged the same neural system as in response inhibition. The Switch condition in the current study was designed to test: (1) whether similar results as reported in Kenner et al. (2010) could be observed without a delay of the response cue; and (2) whether foreknowledge of the presence of the Switch trials would induce similar changes, relative to the Stop condition, in neural activity during the primary “go” responses when the termination of an overt motor response was not expected.

Task Condition was a within-subject factor. All participants were given the three task conditions within an fMRI session. The All-Go condition was always presented first with the Switch and Stop conditions counterbalanced between subjects. All participants were given practice trials at the beginning of the experiment and sufficient time for practicing or getting familiar with the response box. During the experiment, the response box was fixed on the right side of the participant so that it was stationary and no firm hand gripping on the box was necessary.

### Behavioral data analysis

The RT and accuracy data were analyzed using repeated-measures ANOVA (RMANOVA) with Response Type as the repeated measure that included All-Go, StGo (“go” in Stop condition), SwGo (“go” in Switch condition), Switch, and Stop-respond (RT only), planned contrasts, and linear regressions. Prior to data analysis, the RTs of the “go” and the Switch responses that exceeded two standard deviations (SD) of the mean of their respective response type were considered as outliers and replaced by its mean. No more than 5% outliers were identified for any of the response types.

For the Stop condition, SSRT was estimated in two ways (i.e., SSRT_1_ and SSRT_2_). SSRT_1_ was estimated based on the independent “horse-race” model (Logan et al., 1984) that assumes the RT distribution is not affected by the presence of the Stop trials. Under this assumption, SSRT_1_ was derived by subtracting the averaged SSD from the *n*^th^ (where *n* is the percentile corresponding to the probability of the Stop-respond trials) fastest RT of the primary “go” responses (StGo) in the Stop condition (De Jong et al., 1990; Verbruggen and Logan, 2008). SSRT_2_ was estimated with the same method as for SSRT_1_ except that the “go” RT in the All-Go condition was used. If the distribution of the “go” RTs were not or only minimally affected by the presence of the Stop trials, then the relationship between the SSRTs (SSRT_1_ and SSRT_2_) and StGo RT should not change significantly and should not be correlated (Verbruggen and Logan, 2009). The StGo cost was also estimated relative to the “go” RT in the All-Go condition (i.e., [StGo RT—All-Go RT]/All-Go RT). Strong correlation between the SSRTs and StGo cost at the absence of a trade-off in response accuracy would be an indication of a relationship between the primary “go” and the Stop responses.

For the Switch condition, we calculated both the SwGo RT and Switch cost. The Switch cost (i.e., [Switch RT—SwGo RT]/SwGo RT) serves as an index of response switching efficiency, that is, the lower the Switch cost, the more efficient is the Switch response. Similar to the StGo cost, the SwGo cost was estimated by calculating the percent change in the SwGo RT relative to the “go” RT in the All-Go condition (i.e., [SwGo RT—All-Go RT]/All-Go RT). The SwGo cost reflects the additional time needed in making a “go” response knowing the presence of the Switch trials. Linear regression analyses were also carried out between the SwGo RT and Swich cost, and between the SwGo cost and Switch cost. It was predicted that relative to the SwGo RT, the SwGo cost should account for more variance in the Switch cost as it takes into account the “go” RT difference between individuals that was not influenced by the Switch response.

### fMRI data acquisition and analysis

Siemens 3T Verio with a 12 channel head coil. fMRI scans were carried out with gradient echo-planar-Imaging (EPI) sequence: TR = 2000 ms, TE = 25 ms, slice thickness = 4 mm, FOV = 24 cm, design matrix = 64 × 64, flip = 90, and slices = 34. A rear-viewing reflecting mirror was mounted on the MR head coil facing a rear-projection screen placed at the back end of the scanner. A gradient echo EPI fieldmap was acquired for post-scan EPI distortion correction (TR = 1000 ms, TE1 = 3.97 ms, TE2 = 6.43 ms, FOV = 24 cm, slice thickness = 4 mm, design matrix = 64 × 64, flip = 55). T1-weighted anatomical image was acquired using the magnetization prepared rapid gradient echo (MPRAGE) sequence (TR = 3260 ms, TE = 2.26 ms, FOV = 256, slice thickness = 1 mm, design matrix = 256 × 256).

fMRI data were processed and analyzed using the SPM8 software (the Wellcome Department of Imaging Neuroscience, University College London, London, UK). All EPI images were distortion corrected with a gradient echo EPI field-map collected during the fMRI session, and slice-timing corrected, realigned, and coregistered with the subject’s own high resolution T1 anatomical image. The DARTEL software and procedures were used to normalize the T1 and the EPI images to the MNI (Montreal Neurological Institute, Canada) template. At the first level analysis, the design matrix included seven response types (All-Go, SwGo, Switch, StGo, Stop-inhibit, Stop-respond) plus the error response (a nuisance variable), and six motion parameters as separate regressors. The efMRI activation was modeled using the canonical hemodynamic response function (HRF) with temporal and dispersion derivatives. The data were high-pass filtered at 64 s and the epoch/event duration was set at 1 s. Contrasts from the first level individual analysis were fed into the second (group) level analysis using the “One-way within-subject” design and t-tests within the SPM8 software. Sphericity for Subjects was set to be independent and for Response Type dependent. All statistical contrasts were corrected for multiple comparisons and all reported significant voxels survived a corrected multiple-comparison threshold of *p* < 0.05. All activation loci reported in the study were verified using the Anatomy software (Eickhoff et al., 2005, 2007) and the WFU PickAtlas software (by the Functional MRI Laboratory at the Wake Forest University School of Medicine, CA) with the Automated Anatomical Labeling (Tzourio-Mazoyer et al., 2002; Maldjian et al., 2003).

Linear regression analyses were performed with behavioral performance data (i.e., SSRT and Switch cost) and efMRI activation using a binary mask that included a priori regions of interest (ROIs): supplementary motor area (SMA), preSMA, rIFC, left M1, and the basal ganglia. These are critical regions in the FBG inhibitory network. The binary mask was created using the bain atlas (in MNI space) included in the WFU PickAtlas software. The regression analyses examined whether activation during the “go” responses in these critical regions was predictive of response inhibition time (SSRT for the stop-inhibit trials) and cost (for the Switch trials). Significant correlations between the activation in these regions and SSRT or Switch cost would be consistent with previous findings that the “go” process may be influenced by the presence of trials requiring inhibition responses that engage response stages underlying both the “go” and “inhibition” processes (Ko and Miller, 2011, 2014; Ko et al., 2012; Schall and Godlove, 2012). All significant clusters were small-volume (8 mm-diameter) corrected for multiple comparisons (FWE < 0.05).

### Electromyography and data analysis

Electromyography (EMG) of muscle movement was recorded with electrodes placed over the first dorsal interosseous (FDI) in each hand during the fMRI scans using an MRI compatible EMG recording system BrainAmp ExG and software (by Brain Products, Germany). The initial sampling rate was 5 kHz and then, band-pass filtered from 10 to 400 Hz and down sampled to 1 kHz. The EMG data were averaged for each response types (i.e., All-Go, SwGo, Switch, StGo, Stop-inhibit, Stop-respond) around the stimulus onset (segment duration: −100 ms to 2000 ms). For the Stop-inhibit trials, if the EMG amplitude of a trial did not clearly differ from the baseline (i.e., 100 ms prior to the stimulus onset) or exceed 100 uV at the peak within a second after the stimulus onset, it was considered null EMG activity and excluded from averaging. The averaged EMG data were normalized within subject to the baseline for each response type. The EMG onset was defined as >1.5 times of the baseline EMG amplitude measured for each response type, which provided most reasonable estimates of EMG onsets across response types. A RMANOVA was performed to examine differences in EMG onset time (in milliseconds) between response types. One subject’s EMG data was not included in the analysis because of a technical problem during recording.

## Results

### Behavioral results

Separate RMANOVAs were performed for the RT and accuracy data with Response Type as a repeated measure. The RT results (Table 1) showed a significant main effect of Response Type (*F*_(4,68)_ = 64, MSe = 1519, *p* < 0.0001). *Post hoc* multiple comparisons using Tukey test (*p* < 0.05) showed that the RTs of All-Go and Stop-respond trials did not differ statistically from each other, but were significantly faster than that of SwGo, StGo, and Switch responses. The Switch RT was significantly longer than that of SwGo and StGo. RMANOVA with response accuracy showed no statistical difference between the “go” responses in all task conditions (All-Go = 99%, SwGo = 98.8%, and StGo = 98.4%). The lack of significant differences in response accuracy between the “go” responses suggests that the longer RT of SwGo and StGo relative to the All-Go responses cannot be simply attributed to “response-strategy adjustment” (Verbruggen and Logan, 2009), but is likely due to the presence of the Stop or Switch trials in the Stop and the Switch conditions (see further discussion below).

**Table 1:** **Mean RT, accuracy, and EMG onset for all response types in the task conditions**.

Response type	All-Go	SwGo	StGo	Switch	Stop-respond	Stop-inhibit
RT (ms)	585 (56)	658 (80)	652 (77)	761 (80)	578 (50)
ACC (%)	99 (1.6)	98.8 (1.2)	98.4 (1.7)	95.1 (3.8)
EMG onset (ms)	259 (32)	289 (36)	289 (39)	300 (38)	269 (30)	328 (41)

*Note: It shows the mean RT, accuracy, and EMG onset time for all response types in the three task conditions. All-Go = “go” response in the All-Go condition; SwGo = “go” response in the Switch condition; StGo = “go” response in the Stop condition; Switch = Switch responses; Stop-respond = failed-to-stop response in the Stop condition; Stop-inhibit = successfully stopped responses; ( ) = standard deviation*.

**Figure 2:**
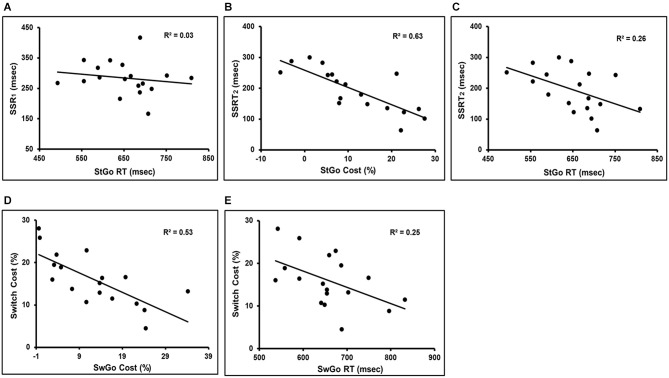
**It shows the linear regression results of the “go” response cost/RT in the presence of the inhibition trials and their relation to the Switch cost and SSRT**. There was no significant correlation between SSRT_1_ and StGo RT (Panel **A**). Panels **(B,C)** are the regression results between the SSRT_2_ and the StGo cost or StGo RT. Panels **(D,E)** show the regression results between the Switch cost and the SwGo cost or SwGo RT. SwGo = the “go” trials in the Switch condition; StGo = the “go” trials in the Stop condition; SSRT_1_ = stop-signal response time; SSRT_2_ = adjusted stop-signal response time.

On average, SSRT_1_ (based on the StGo RT distribution) = 284 ms, and SSRT_2_ (based on the All-Go RT distribution) = 194 ms, SSD = 327 ms (± 63), estimated Stop-respond RT = 553 ms, the observed Stop-respond RT = 578 ms. To further examine whether the presence of the Stop and Switch trials influenced the StGo and SwGo RTs and its relationship to the SSRT, the estimated SSRTs were fitted in separate linear regression analyses with the StGo RT and StGo cost. There was no significant correlation between SSRT_1_ and the StGo RT (Figure 2A). This was consistent with previous findings that the RT of the primary “go” responses is not correlated with the Stop inhibition time (or SSRT) when the SSRT was estimated based on the “go” responses within the same task condition. An RMANOVA with the variances from the All-Go, SwGo, and StGo RT (i.e., RT Type) as the repeated measures showed a significant effect of RT Type (*F*_(2,34)_ = 16.6, MSe = 1.336E + 07, *p* < 0.0001). *Post hoc* Scheffe contrasts (*p* < 0.05) showed that both the variance of the SwGo RT (1.100E + 04) and of the StGo RT (1.204E + 04) were significantly larger than the All-Go RT (5.511E + 03), indicating a change in the RT distribution when the Stop or the Switch trials were present in the task set.

However, significant negative correlations were observed between SSRT_2_ and StGo RT or StGo cost (with StGo RT: *t*_17_ = −2.34, *p* < 0.05, *R*^2^ = 0.26; with StGo cost: *t*_17_ = −5.16, *p* < 0.0001, *R*^2^ = 0.63) when the estimation of SSRT was based on the All-Go RT distribution (Figures 2B,C). The correlation between SSRT_2_ and StGo cost was much stronger and accounted for more variance than that between SSRT_2_ and StGo RT. A similar significant linear correlation remained even when only a subset of nine subjects with a percentage of stop-inhibit responses much closer to the 50% range (40–55%) were included (with StGo cost: *t*_8_ = −2.73, *p* < 0.03, *R*^2^ = 0.52; not significant with StGo RT). There was no significant correlation between the estimated RT of the Stop-respond trials and the percentage of the Stop-respond trials, indicating that the difference between the estimated and observed Stop-respond RT was not due to a subset of subjects with higher or lower percentage of Stop-respond trials.

To examine the relationship between the Switch cost and the SwGo RT, simple linear regression analyses were performed with either the SwGo RT or cost (see Method for estimating SwGo cost) as the predictor variable. The results showed a significant negative correlation between the SwGo cost and Switch cost (*t*_17_ = −4.25, *p* < 0.001, *R*^2^ = 0.53) (Figure 2D). As the SwGo cost increased, the Switch cost decreased or Switch responses became more efficient. Similar regression analysis using the absolute SwGo RT as a predictor showed a weaker correlation with the Switch cost and accounted for much less variance (*t*_17_ = −2.32, *p* < 0.05, *R*^2^ = 0.25) (Figure 2E). These results suggest that changes in Switch cost or Switch efficiency was not simply due to a shift or delay in the absolute RTs for all responses, but related specifically to the changes in the SwGo response process when the Switch trials were present.

### efMRI activation and its correlation with behavioral measures

**Figure 3:**
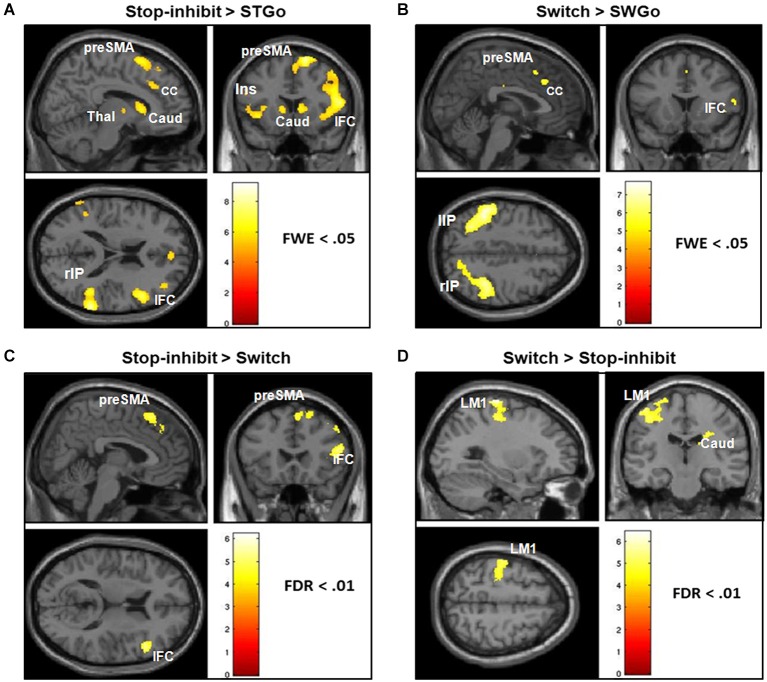
**It shows the results of whole-brain analysis of fMRI BOLD activation of responses associated with the Switch and successfully stopped (Stop-inhibit) responses**. Panels **(A,B)** show the differential activation of the Stop-inhibit and Switch responses after subtracting out activation associated with the “go” responses. Panels **(C,D)** show brain regions that had significantly stronger activation during the Stop-inhibit responses relative to Switch, and during the Switch relative to the Stop-inhibit responses. StGo = “go” responses in the Stop condition; SwGo = “go” responses in the Switch condition; FDR = error correction using the topological false-discovery rate.

**Figure 4:**
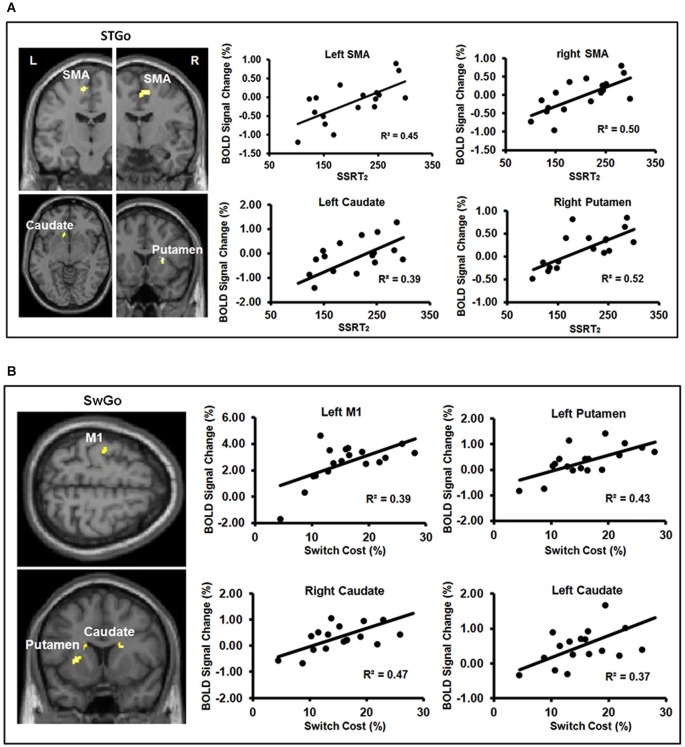
**(A,B)** show the results of linear regressions between behavioral measures of inhibition efficiency and percent BOLD signal change during the “go” responses. Panel **(A)** shows significant linear relations between the behavioral measure ( SSRT_2_) of Stop efficiency and BOLD signal change in the FBG regions associated with the “go” responses in the Stop condition. Panel **(B)** shows significant linear relations between the Switch cost/efficiency and BOLD signal change in the FBG regions associated with the “go” responses in the Switch condition. M1 = primary motor cortex; SMA = supplementary motor area. (Note: One subject’s data was not included in Panel **(A)** because of an unusually short SSRT_2_ (64 ms) to avoid inflating the regression coefficient).

Consistent with previous findings, a whole-brain analysis showed that relative to the StGo in the Stop condition and the Switch responses, Stop-inhibit trials induced significantly more activation in the right pre-SMA, rIFC, insula, putamen, caudate, anterior cingulate, and the inferior parietal cortex (IPC; Figures 3A,C; Table 2). ROI analyses further revealed significantly more activation in the pallidum and sub-thalamic nucleus during Stop-inhibit relative to StGo responses (Table 2). In addition, the regression analysis showed a significant correlation (FWE < 0.05) between the SSRT2 and activation during StGo responses in the following brain regions that are critical part of the FBG network: left and right SMA; left caudate and right putamen (Figure 4A).

**Table 2:** **Significantly activated brain regions during Stop-inhibit and Switch responses**.

Response and contrast type	L/R	Brain regions	x y z	Cluster size
**Stop-inhibit > StGo** (Whole brain)	R	IPC (BA 40, 39; angular, supra marginal)	64, −44, 14	9762
	L	IPC (BA 40, 39; angular, supra marginal)	−54, −42, 39	4197
	R	IFC (BA 44, 45; opercularis, middle frontal, insula)	54, 16, 15	7508
	L	IFC (BA 44; opercularis)	−50, 12, 26	64
	L	Insula	−32, 18, 6	836
	R	Caudate	−12, 18, 5	349
	L	Caudate	−12, 12, 6	226
	R	Thalamus	10, −6, 5	21
	R	Middle temporal (BA 39)	48, −20, −7	21
	R	Pre-SMA (BA 6)	16, 13, 66	3342
		Anterior cingulate (BA 32)	3, 49, 17
		Middle cingulate (BA 24)	4, 28, 38
	R	Middle frontal (BA 9)	24, 52, 32	437
	L	Middle frontal (BA 9, 10)	−40, 34, 36	127
**ROIs:**	R	Pre-SMA (BA 6)	16, 13, 66	2289
		Left Pre-SMA (BA 6)	−8, 19, 50
	R	IFC (BA 44, 45; Opercularis)	54, 16, 15	2502
	R	Caudate	12, 18, 5	1684
		Putamen	28, 19, 2
		Pallidum	18, 9, 3
	L	Caudate	−12, 12, 6	1292
		Putamen	−20, 15, 9
		Pallidum	−12, 6, 3
	L	Sub-thalamic nucleus	−8, −12, −6	31
**Switch > SwGo** (Whole brain)	R	(IPC (BA 40, 39; supra marginal)	48, −41, 41	2950
	L	IPC (BA 40, 39; supra marginal)	−45, −42, 48	3258
	R	SPC (BA 7)	30, −62, 57	334
	L	SPC (BA 7)	−32, −57, 60	533
	R	IFC (BA 44; opercularis)	55, 13, 12	37
	R	IFC (BA 44, 45; triangularis, orbitalis, insula)	52, 21, 0	373
	R	Pre-SMA (BA 6)	3, 18, 48	24
	R	Middle cingulate (BA 23, 31)	2, −21, 30	24
	R	Middle frontal (BA 9)	30, 6, 58	156
	L	Middle frontal (BA 9)	−32, 7, 65	28
**ROIs:**	R/L	Pre-SMA (BA 6)	3, 18, 48	880
		Pre-SMA (BA 6)	−3, 22, 44
	R	Pre-SMA (BA 6)	16, 13, 63	386
	R	IFC (BA 44, 45; opercularis)	55, 13, 12	1762
	L	LM1 (BA 4)	−54, −17, 32	136
			−44, −14, 59	28
	R	Caudate	10, 19, −1	936
		Putamen	20, 12, −10
	L	Putamen	−18, 0, 11	90
		Putamen	−20, 3, −10	38
**Stop-inhibit > Switch** (Whole brain)	R	Middle frontal (BA 8, 9)	39, 31, 48	874
	R	SFC (BA 9)	26, 49, 39	181
	R	IFC (BA 44, 45)	52, 21, 15	498
	R	IPC (BA 40, 39; supra marginal, angular)	58, −51, 32	126
	R	Pre-SMA (BA 6; superior medial gyrus)	6, 25, 57	892
	R	Insula	37, 24, 5	18
	R	Occipital (BA 17, 18)	28, −92, −12	121
	R	Occipital (BA 17, 18)	−26, −98, −9	52
**Switch > Stop-inhibit** (Whole brain)	L	M1 (BA 4a, 4p)	−32, −26, 70	783
	L	Postcentral (BA 1, 2, 3)	−40, −21, 51	743
	R	Cerebellum (VI)	21, −57, −24	165
		Cerebellum (VIII)	28, −48, −54	13
	R	Postcentral (BA 3b)	64, 0, 17	41
	L	Superior Occipital (BA 19)	−15, −90, 24	14
**Common activation of Stop-inhibit and and Switch** (Whole brain)	R	Pre-SMA	3, 18, 48	21
	R	RIFC (BA 44, 45; triangularis, opercularis)	52, 21, 0	243
	R	Insula	37, 20, 0	161
	R	Mid cingulate cortex (BA 32)	3, 27, 36	63
	R	Middle frontal	33, 7, 57	43
	R	IPC (BA 40, 39; supra marginal, angular)	48, −41, 41	2160
	L	IPC (BA 40, 39; supra marginal, angular)	−52, −39, 39	1952

*Note: It shows significantly activated brain regions during Stop-inhibit and Switch responses using both the whole brain analysis and a priori regions of interest (ROI) analyses. SwGo = “go” trials in the Switch condition; StGo = “go” trials in the Stop condition; Stop-inhibit = successfully stopped trials; L/R = left or right; BA = Brodmann’s areas; IPC = inferior parietal cortex; coordinates (x y z) are in MNI space; SPC = superior parietal cortex; IFC = inferior frontal cortex; Pre-SMA = pre-supplementary motor area; SMA = SMA proper; SFC = superior friontal cortex; M1 = primary motor cortex; ROIs = regions of interest (FDR corrected with p < 0.01)*.

The Switch response showed much weaker activation in the rIFC relative to the Stop-inhibit (Figure 3D). This was the case even when the Switch response was contrasted with SwGo (Figure 3B). A priori ROI analysis showed that, relative to the SwGo responses, Switch had significantly more activation in the pre-SMA, right IFC, caudate, and putamen of the basal-ganglia (Table 2). A whole-brain conjunction analysis (Friston et al., 1999; Nichols et al., 2005) examining the shared regions of activation during the Switch and Stop-inhibit responses further revealed significant common activation in several regions including the right preSMA, rIFC, insula, anterior cingulate, and the bilateral IPC (Table 2). These results were consistent with previous findings that stopping and switching responses engage a common inhibitory system (Kenner et al., 2010). In addition, the linear regression analysis with the Switch cost also showed that activation of several regions during the SwGo responses was highly correlated with the Switch cost (Figure 4B). The Switch cost, but not the Switch RT *per se*, was positively correlated (FWE < 0.05) with activation during the SwGo responses in the LM1, left putamen, right and left caudate.

### EMG results

RMANOVA showed a significant main effect of EMG onset time (*F*_(5,80)_ = 21.2, MSe = 480.3, *p* < 0.0001) (see Table 1). *Post hoc* multiple comparisons using Least Significant Difference at *p* < 0.05 showed that the EMG onsets of All-Go (259 ms) and Stop-respond (269 ms) trials did not differ significantly from each other, but were significantly earlier than that of SwGo (289 ms), StGo (289 ms), and Switch (300 ms). There was also no statistical difference in EMG onset between Switch, SwGo, and StGo. Eighty-five percent of the Stop-inhibit trials also showed clear EMG activity (see criteria in Section Methods) that was coupled with stimulus onset. However, the EMG onset (328 ms) of Stop-inhibit trials was significantly later than the other five types of responses, but was virtually identical to the averaged SSD time (327 ms), indicating a reactive component of the Stop-inhibit response close to the stop-signal onset. Relative to the StGo responses, the EMG onset of the Stop-inhibit trials was about 39 ms later, and its averaged peak EMG amplitude (106, in % change relative to the baseline) was 77.3% of the amplitude (130.7) of the All-Go trials.

## Discussion

Behavioral results of the present study showed that the presence of Switch or Stop trials coincided with a significant increase in the cost of the primary “go” (SwGo and StGo) responses. In addition, when the SSRT estimate was based on the All-Go RT distribution that was not influenced by the presence of Stop trials, a significant negative correlation was observed between the SSRT_2_ and StGo RT as well as with the StGo cost. As the StGo RT and StGo cost increased, SSRT_2_ decreased. The negative correlation was stronger and accounted for more variance when SSRT_2_ was correlated with StGo cost than with the StGo RT directly. Significant correlation between the SSRT and the StGo cost is indicative of a relationship between the efficiency of the primary “go” and Stop responses. Similar correlation was also observed in the Switch condition where significant negative correlation between the Switch cost and SwGo cost accounted for more than 50% of the variance relative to that between Switch cost and SwGo RT directly. These results suggest that knowing the presence of the Stop and Switch trials prior to the task has a global influence on the primary “go” response process which in turn, modulates the efficiency of rapid response inhibition both in stopping an overt on-going motor response (as for the Stop trials) and in stopping an impulse or tendency for the primary “go” response (as for the Switch trials).

Consistent with the behavioral results, efMRI activation data also showed that neural activity during the “go” (StGo and SwGo) responses varied with the inhibition time ( SSRT_2_) of Stop responses and with the Switch cost. In several of the a priori brain regions (i.e., LM1, SMA, pre-SMA, caudate, and putamen) of the FBG network, efMRI activation coupled with the StGo and SwGo responses was significantly correlated with the critical behavioral measures (i.e., SSRT_2_ and Switch cost) of the efficiency of Stop and Switch responses. These results are consistent with recent findings that fMRI activation in regions of the FBG network during the Stop responses correlated with the “go” RT cost with trial-by-trial cues (Chikazoe et al., 2009; Jahfari et al., 2010). They are also consistent with the predictions that knowing the presence of inhibition trials prior to the task is sufficient to influence neural activity associated with the primary “go” response which, in turn, modulates the efficiency of rapid inhibition responses, irrespective of whether the termination of an overt response is required.

Unlike previous studies using trial-by-trial pre-stimulus cues or very short blocks of trials, the current study design focused on the effect of the foreknowledge prior to the task and included averaged brain activation of all “go” responses. Therefore, the significant correlations were unlikely just due to the “go” response immediately preceding a Stop or a Switch trial, but a more general dynamic relationship between neural activity gearing up for a “go” response while anticipating to rapidly terminate it when needed. Furthermore, efMRI activation during the “go” responses in both the Stop and Switch conditions showed significant correlations with the response inhibition time (SSRT_2_) and Switch cost in the striatum regions of the FBG network. Evidence of a coupling between the activity in the striatum during the “go” responses and the efficiency of the Stop or Switch responses suggests that foreknowledge/context influences not only the peripheral motor system (e.g., M1), as reported in previous studies (Cai et al., 2011; Ko and Miller, 2011; Ko et al., 2012), but more extensively the FBG network as a whole. This dynamic relationship between the neural activity during the primary “go” response and the cost in Stop and Switch RTs is likely due to a shared response system of which the neural activity for a “go” response and its ability to stop it, when needed, may be interdependent. The “go” response cost in the Stop and the Switch conditions is likely, at least in part, a result of such dynamic control process.

As reported in previous studies, the “go” response cost in the Stop condition cannot be simply attributed to a deliberate response-delay at the trial level (Chikazoe et al., 2009; Verbruggen and Logan, 2009; Jahfari et al., 2010) because this strategy reflects top-down control which is a relatively slow process and appears to have little involvement during online execution of mental operations (Posner, 1980; Ruthruff et al., 2001). Our results showed that the average RT of the StGo and SwGo differed from the All-Go RT by less than 80 ms, but the observed mean SSD in the Stop condition was about 327 ms. If subjects deliberately delayed their responses for all trials in order to catch the stop-signal or to have sufficient cue-encoding time, much larger differences in reaction time, at least, for the StGo should be observed. However, this is not the case, and the StGo RT was virtually identical to the SwGo RT in the Switch condition that had no delay in Switch cues. In addition, the EMG data showed that both the StGo and SwGo responses had an EMG onset of about 289 ms after the stimulus onset, and that the EMG onset for the Stop-inhibit trials that showed EMG activity was about 328 ms. These data suggest that the “go” process must have started much earlier than the SSD and that the “go” response cost could not be simply attributed to a voluntary response-delay strategy at the presence of SSDs. The response cost of the “go” trials also cannot be explained by the “response-strategy adjustment” hypothesis that participants raise their response threshold to increase the opportunity for making a correct response or rapid stopping when stop-signal occurs, and therefore the slowing of the “go” process affects only response execution and not the inhibition process *per se* (Verbruggen and Logan, 2009). The “response-strategy adjustment” hypothesis predicts a speed-accuracy trade-off as it was reported in Verbruggen and Logan (2009) study. However, no apparent speed-accuracy trade-off was observed in the current study. Instead, consistent with previous studies (Hester et al., 2004; Fassbender et al., 2009), our results suggest that knowing the presence of inhibition responses, the top-down influence likely started earlier on the task-set level (Lien et al., 2005) such that it conditioned task-relevant neural activity for an optimal probability of making a stopping or switching response.

Although the exact mechanism contributing to the “go” response cost cannot be determined with the current experimental design, we postulate that a large component of the cost involved an early inhibitory process in anticipation for a Stop and Switch response. Previous studies have shown that the anticipatory (or preparatory) process engaged task-relevant brain regions (Apicella et al., 1992; Schultz et al., 1992; Hester et al., 2004; Zandbelt and Vink, 2010) and that at the presence of stop-signal trials, selective reduction of neural excitability in response-specific motor cortex occurred prior to the stimulus onset (Claffey et al., 2010; Jahfari et al., 2010; Cai et al., 2011; Greenhouse et al., 2012; Majid et al., 2012). Schultz et al. (1992) showed that when an instruction indicated a “no-go” response for an upcoming trial, neuronal impulse (firing) in the ventral striatal region was significantly reduced relative to when the instruction indicated a “go” (or movement) response (also see Apicella et al., 1992). Jahfari et al. (2010) and Cai et al. (2011) showed a significant reduction in neural excitability in the primary motor cortex prior to stimulus onset when subjects were given prior warning for stop responses in some of the upcoming trials. Our results further support an early inhibitory process beyond the motor cortex and in a task condition (such as the Switch condition) that did not require the termination of an overt motor response. In agreement with previous findings, the “go” response cost in this study is, at least in part, due to a process modulating the effector and action specific neural activity and gearing up the system (e.g., activity in the FBG network) for a potential Stop or Switch response. Consequently, the efficiency of the inhibition response may be influenced by the dynamic modulation of neural activity underlying the primary “go” process. Such neural modulation may be triggered automatically when foreknowledge about the presence of inhibition trials is given.

In summary, the results of the current study suggest that knowing the presence of inhibition trials prior to the task is sufficient to influence neural activity associated with the primary “go” responses, which, in turn, may modulate the efficiency of rapid inhibition, irrespective of whether the termination of an overt response is required. This dynamic relationship between the primary “go” response and the efficiency of rapid response inhibition is likely modulated by the activity in the FBG network beyond the peripheral motor system.

## Conflict of interest statement

The authors declare that the research was conducted in the absence of any commercial or financial relationships that could be construed as a potential conflict of interest.

## Footnotes

^1^Here, we loosely define “inhibition trials” as any trials that require the termination/suppression of the primary “go” response (also see Kenner et al., 2010).
